# Growth and Gastrointestinal Tolerance in Healthy Term Infants Fed Milk-Based Infant Formula Supplemented with Five Human Milk Oligosaccharides (HMOs): A Randomized Multicenter Trial

**DOI:** 10.3390/nu14132625

**Published:** 2022-06-24

**Authors:** John Lasekan, Yong Choe, Svyatoslav Dvoretskiy, Amy Devitt, Sue Zhang, Amy Mackey, Karyn Wulf, Rachael Buck, Christine Steele, Michelle Johnson, Geraldine Baggs

**Affiliations:** Abbott Nutrition, Columbus, OH 43219, USA; yong.choe@abbott.com (Y.C.); svyatoslav.dvoretskiy@abbott.com (S.D.); amy.devitt-maicher@abbott.com (A.D.); sue.zhang@abbott.com (S.Z.); amy.mackey@abbott.com (A.M.); karyn.wulf@abbott.com (K.W.); rachael.buck@abbott.com (R.B.); christine.steele@abbott.com (C.S.); michelle.johnson@abbott.com (M.J.); geraldine.baggs@abbott.com (G.B.)

**Keywords:** five human milk oligosaccharides, 2′-FL, 3-FL, LNT, 3′-SL, 6′-SL, infant growth, gastrointestinal tolerance

## Abstract

*Background*: Five of the most abundant human milk oligosaccharides (HMOs) in human milk are 2′-fucosyllactose (2′-FL), 3-fucosyllactose (3-FL), lacto-N-tetraose (LNT), 3′-sialyllactose (3′-SL) and 6′-sialyllactose (6′-SL). *Methods*: A randomized, double-blind, controlled parallel feeding trial evaluated growth in healthy term infants fed a control milk-based formula (CF; n = 129), experimental milk-based formula (EF; n = 130) containing five HMOs (5.75 g/L; 2′-FL, 3-FL, LNT, 3′-SL and 6′-SL) or human milk (HM; n = 104). *Results*: No significant differences (all *p* ≥ 0.337, protocol evaluable cohort) were observed among the three groups for weight gain per day from 14 to 119 days (D) of age, irrespective of COVID-19 or combined non-COVID-19 and COVID-19 periods. There were no differences (*p* ≥ 0.05) among the three groups for gains in weight and length from D14 to D119. Compared to the CF group, the EF group had more stools that were soft, frequent and yellow and were similar to the HM group. Serious and non-serious adverse events were not different among groups, but more CF-fed infants were seen by health care professionals for illness from study entry to D56 (*p* = 0.044) and D84 (*p* = 0.028) compared to EF-fed infants. *Conclusions*: The study demonstrated that the EF containing five HMOs supported normal growth, gastrointestinal (GI) tolerance and safe use in healthy term infants.

## 1. Introduction

Human milk oligosaccharides (HMOs) are non-digestible carbohydrates found in human milk. Their importance to infant nutrition is underscored by their position as the third most abundant solid component of human milk, behind lactose and lipids [1]. HMOs can be structurally categorized as (a) fucosylated HMOs such as 2′- and 3- fucosyllactose (2′-FL and 3-FL), (b) acetylated HMOs such as lacto-*N*-tetraose (LNT) and (c) sialylated HMOs such as 3′- and 6′ sialyllactose (3′-SL and 6′-SL) [1]. The profiles of HMOs in human milk can vary widely due to various influences such as genetics, stage of lactation and geography [2]. While most HMO concentrations decrease over the course of lactation, at least two, 3′-SL and 3-FL, have been reported to increase [3]. Structural differences in HMOs facilitate functional synergy between different categories, as different HMOs may work in cumulative and complementary ways to promote digestive health, immune support and cognitive development [1].

The digestive benefits of HMOs span across three general areas: prebiotic effects, generation of beneficial microbial metabolites and providing gut barrier protection [1]. HMOs act as prebiotics by being selectively utilized by the infant gut microbiota; for example, *Bifidobacteria* spp. and *Bacteroides* spp. use 2′-FL, 3-FL and 6′-SL as substrates for growth [4], and LNT can be utilized as a carbohydrate source for *B. infantis* [5]. These bacteria use HMOs to generate beneficial metabolites such as acetate, propionate and butyrate that have been shown preclinically to exert multiple beneficial physiological effects such as acting as anti-inflammatory agents, serving as energy substrates for intestinal epithelial cells and promoting gastrointestinal (GI) motility [1,6,7]. Preclinical research details the role that HMOs play in gut barrier protection through direct action on the intestinal mucosa. One study demonstrated that a combination of fucosylated, sialylated and acetylated HMOs at levels similar to human milk enhanced gut barrier integrity [8].

HMOs are reported to have immune effects in multiple ways including, anti-adhesive activity and modulation of the immune system [1]. For example, preclinical research shows that 2′-FL may block pathogen adhesion for *Campylobacter jejuni* and Enteropathogenic *E. coli* [9], 3′-SL may block pathogen adhesion for *Pseudomonas aeruginosa* and *Vibrio cholerae* toxin [10,11], and LNT may block pathogen adhesion for *Entamoeba histolytica* and Group B *Streptococcus* [12,13]. Furthermore, preclinical research suggests that HMOs play an important role in the modulation of the immune system, as 3′-SL and 6′-SL have been shown to stimulate chemokine expression and immune cell recruitment to sites of infection [11]. These immune benefits have been shown clinically as well, as the consumption of HMOs has been associated with decreased frequency of respiratory and GI illness in breastfed infants [14]. In a clinical study, breastfed infants and infants fed either of the experimental formulas with 2′-FL had lower concentrations of plasma inflammatory cytokines than did infants fed the control formula without HMOs [15]. The role of HMOs in cognitive development is an exciting new area of research. Breastfeeding association studies have shown the benefits of HMOs, and higher levels of 2′-FL and 6′-SL in breastmilk have been linked with cognitive development through 24 months of age [16,17,18]. 

Studies have shown that supplementing infant formulas with commercially available HMOs, 2′-FL [19,20], and 2′-FL and LNnT [21] was safe, well-tolerated, supported age-appropriate growth and provided immune benefits, including fewer reported respiratory infections and bronchitis [19,20,21]. Furthermore, a recent clinical study demonstrated that the combination of five HMOs (2′-FL, 3-FL, LNT, 3′-SL and 6′-SL) in infant formula was safe, well tolerated and supported normal growth and development in healthy term infants [22]. Improving the HMO profile of infant formulas to be more like that of human milk can be explored in a continued effort to bridge the gap between human milk-fed and formula-fed infants. Therefore, the purpose of this study was to evaluate the growth and GI tolerance of infants fed a novel milk-based infant formula supplemented with a blend of five commercially available HMOs in concentrations within the range found in human milk. HMOs were added to be within human milk ranges, 2′-FL at 3.0 g/L (0.004–6.00 g/L in human milk), 3-FL at 0.8 g/L (0.023–6.08 g/L in human milk), LNT at 1.50 g/L (0.003–2.76 g/L in human milk), 3′-SL at 0.20 g/L (0.026–0.48 g/L in human milk) and 6′-SL at 0.30 g/L (0.011–1.57 g/L in human milk) [23,24,25,26,27,28,29]. These were based on the average of the ranges and proportions of these five HMOs in human milk reported in seven independent studies [23,24,25,26,27,28,29]. These HMOs are among the most abundant HMOs typically found in most mothers’ milk and represent different structural classes, each responsible for unique benefits for the infant [1,30].

## 2. Materials and Methods

### 2.1. Study Design

**Figure 1:**
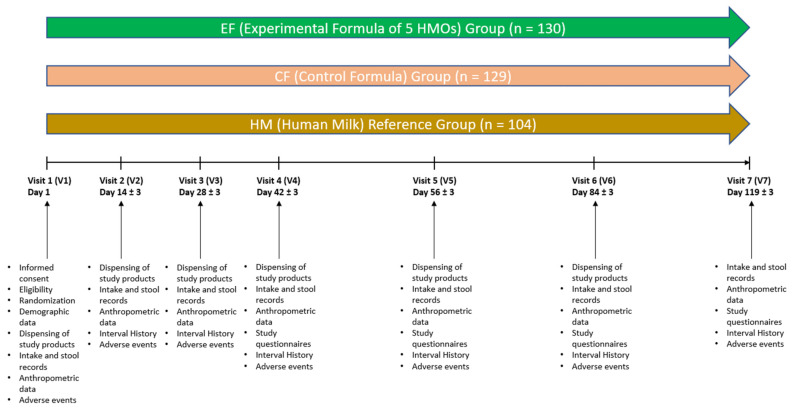
Study flowchart.

This randomized, controlled, multicenter, double-blind, parallel feeding growth and tolerance study was conducted at 34 study sites throughout the United States from September 2019 through December 2020, mostly during the COVID-19 pandemic when stay-at-home orders were in place. Healthy, full-term infants were enrolled at ≤14 Days (D) of age. Formula-fed infants who met the eligibility criteria were randomized to receive either a control (CF) or an experimental formula (EF) through approximately 4 months of age (Figure 1). The CF contained no added HMOs or other prebiotics. The EF was nutritionally similar to the CF and was supplemented with a blend of 5 HMOs. A nonrandomized human milk (HM)–fed group was also included. Parents were asked to feed the assigned study formula or human milk as their infants’ sole source of nutrition until D119 or up to D183 during the COVID-19 pandemic. The study protocol, informed consent forms (ICF), applicable privacy regulation authorization, recruitment materials, and any written information provided to the participant’s parent(s) were reviewed and approved by the Copernicus Group Independent Review Board of the Western Institutional Review Board (WCG IRB), Puyallup, WA, USA. The study was performed in accordance with the protocol and the consensus ethical principles derived from international guidelines, including the Declaration of Helsinki, and applicable ICH Good Clinical Practice (GCP) Guidelines. The study was registered at clinicaltrials.gov (Registration # NCT04105686).

### 2.2. Participants

Participants were eligible for the study if they were singleton, healthy term infants (gestational age 37–42 weeks) between 0 and 14 days of age at enrollment, with a birth weight ≥ 2490 g. Participants were also eligible for the study if judged to be in good health, as determined from the infant’s medical history and parental report. Participants whose parents had voluntarily confirmed their intention to feed the assigned study formula or human milk (HM) as the sole source of nutrition for the duration of the study unless instructed otherwise by their health care professionals were enrolled into the study. Enrolled participants included those whose parent(s) had voluntarily signed and dated an ICF, approved by the IRB/IEC and provided Health Insurance Portability and Accountability Act (HIPAA) (or other applicable privacy regulation) authorization prior to any participation in the study. Participants were excluded from the study if they had maternal, fetal or perinatal medical conditions with potential adverse effects on GI tolerance, growth and development, including suspected substance abuse. Gestational diabetes was acceptable if the infant’s birth weight was equal to or less than the 2009 Centers for Disease Control Growth Charts (2006 WHO) 95th percentile [31]. Participants were also excluded from the study if they were taking medications (including over the counter (OTC) medications such as Mylicon^®^ for gas), prebiotics, probiotics, home remedies (such as juice for constipation), herbal preparations or rehydration fluids that might affect GI tolerance.

### 2.3. Study Treatments

Infants in the study were fed a control or experimental cow’s-milk-based infant formula, or human milk. Both study formulas contained 676 kcal/L (20 kcal/fl oz), and their composition was similar to that of a commercial milk-based formula. The control product was a milk-based formula without HMOs or other prebiotics. The experimental formula was similar to the control formula except it contained a blend of 5 HMOs at the following concentrations: 3.0 g/L of 2′-FL, 0.8 g/L of 3-FL, 1.5 g/L of LNT, 0.2 g/L of 3′-SL and 0.3 g/L of 6′-SL. Both study formulas contained 14 g/L of protein, 36.5 g/L of fat and 74 g/L of carbohydrate. Infants in the HM-fed group were fed mother’s milk by breast or bottle. Parents were instructed to feed the assigned formulas *ad libitum*. All study formulas met the nutrient requirements of the Federal Food Drug and Cosmetic Act, Section 412 (350a) [32] and its corresponding regulations, 21 CFR 107.100 [33]. The formulas were similar in appearance, consistency and odor. Formulas were provided as ready-to-feed liquids in 32 fl oz re-closeable plastic bottles, and the bottles were clinically labeled with a product code to mask the identity of the formulas.

### 2.4. Study Outcome Measures and Procedures

The primary outcome for this study was weight gain per day, from D14 to D119. Secondary variables were weight, interval weight gain per day, length, interval length gain per day, head circumference (HC) and interval HC gain per day. Meanweight for age, mean length for age and mean HC for age were plotted on the WHO growth charts. [31]. Mean rank stool consistency (MRSC), number of stools per day and percentage of feedings with spit-up/vomit associated with feeding (within 1 h) were secondary outcomes. Supportive variables included additional infant and maternal demographics, formula intake, and parental responses to questions related to their satisfaction with the formula and their infant’s behavior.

At enrollment, eligible participants were randomized to one of the study formula groups or enrolled in the HM group. Randomization schedules were computer-generated using a dynamic minimization algorithm with a random component. The electronic data capture system was used to assign unique participant numbers and randomize participants to study product codes according to the generated randomization schedules. Randomization was stratified by sex. A central randomization approach was utilized. In addition, demographic information and anthropometrics (birth weight, length and HC) were obtained from infants’ parents at enrollment. After enrollment, infants were seen at study visits at D14, D28, D42, D56, D84 and D119. The D14, D28, D42, D84 and D119 visits had a window of ±3 days. At each study visit interval, clinical and diet histories were obtained and recorded, and anthropometric assessments of participants were conducted. Parents of formula-fed infants were supplied with enough assigned formula to last until the next visit.

Weight, length and HC were measured at all study visits using standard methods [34,35]. Infants were weighed twice unclothed on calibrated electronic scales. All anthropometric measurements were performed twice. A third measurement was required if the difference between the first two measurements exceeded the defined limits, which were >10 g for weight, >0.4 cm for length and >0.2 cm for HC. Infant length was measured with the infant in a recumbent position using a pediatric length board. HC was measured around the occipital frontal area using the flexible, non-stretchable measuring tape supplied by the sponsor. A training guide explaining procedures to obtain anthropometric measurements was provided.

In response to the COVID-19 pandemic, the protocol was amended to allow for alternative anthropometric measurements and extend the study visit window to complete the last study visit (D119) up to 183 days for those participants who were unable to meet the visit requirements [36]. During this extended period, participants were to continue their assigned study feeding as sole source of nutrition.

Alternative anthropometric measurements included weight, length and HC measurements obtained from well-baby or sick visits by non-study health care professionals (HCPs) or weight measurements by parents/caregivers. Parents who provided in-home weight measurements were trained to obtain and record three weight measurements using the calibrated electronic scale supplied by the study sponsor. Anthropometrics were plotted on the 2006 WHO growth charts at each of the study visits. Parents were not asked to obtain length and HC measurements since these measurements are difficult to obtain.

Study intake and stool records, including the volume of formula consumed at each feeding or the number of HM feedings, incidence of spit-up and vomiting associated with feedings, and infant’s stool characteristics (frequency, consistency and color) were collected by parents and reviewed by study staff at each visit to ensure they were completed correctly and thoroughly. Parents of participants also completed infant feeding and stool patterns questionnaires (at D42, D84 and D119), infant behavior questionnaires (D56 and D119) and formula satisfaction questionnaires (formula-fed group only) at the D119 visit using questionnaires validated and used in published pediatric clinical studies [37,38]. The consumption of human milk/formula other than the assigned study feeding as well as the use of medications, supplements, home remedies or other sources of nutrition were monitored and recorded throughout the study.

Non-serious adverse events (NSAEs) and serious adverse events (SAEs) were medically confirmed and assessed for causality by the study physician. Adverse events (AEs) were coded and grouped according to the Medical Dictionary for Regulatory Activities (MedDRA) by primary system organ class (SOC) and preferred terms (PT).

### 2.5. Statistical Analyses

The primary analysis for this study included only data from those participants determined to be protocol-evaluable (PE). A secondary analysis and the safety data analysis included all available data (ITT) from participants who received at least one feeding of the study formula during the study period. Models including only EF and CF were the primary comparisons of interest. Models including the HM reference group were also evaluated and considered secondary comparisons of interest.

A sample size of 64 participants in each study group yielded 80% power to detect a difference in means of at least 3 g/d when the standard deviation was 6 g/d for the primary variable, weight gain per day from D14 to D119, using a two-group *t*-test with a 0.05 two-sided significance level (nQuery Advisor^®^ Version 7.0). A total of 366 participants were ultimately enrolled to account for attrition and protocol deviations during the COVID-19 pandemic.

The primary study variable, which was weight gain per day from D14 to D119, was analyzed using an analysis of covariance (ANCOVA) with factors of study center, sex, study group, study group by sex interaction and covariate birth weight. The upper limit of a one-sided 97.5% confidence interval of the difference in least squares means (µCF − µEF) generated from the ANCOVA model was evaluated to detect non-inferiority. In this trial, non-inferiority (using an equivalence limit of 3 g/d as per the American Academy of Pediatrics, Committee on Nutrition recommendation of a nutritionally significant difference [39]) was tested using the following hypotheses:

H0 (null): µCF − µEF ≥ 3.0 (EF inferior to CF) vs. HA (alternative): µCF − µEF < 3.0 (EF not inferior to CF).

The Gompertz model [40] was used to describe the growth curve for each individual infant, and values at D14, D28, D42, D56, D84 and D119 (whether missing or non-missing) predicted from the Gompertz fit were used for this primary analysis. Sensitivity analyses included the evaluation of observed weight gain per day from D14 up to D183 including only the site collected, as well as alternatively collected (parent and/or non-study HCP) anthropometric measurements obtained during the pre-COVID-19 and post-COVID-19 periods combined and the post-COVID-19 period only.

All tests of hypotheses were two-sided, 0.05-level tests, except tests for interaction effects, which were two-sided, 0.10-level tests, and tests for non-inferiority, which were one-sided, 0.025-level tests. An adjustment to the α-level for multiple comparisons was made using Holm’s step-down Bonferroni procedure. Continuous variables (anthropometric, intake and stool outcomes) were evaluated using analysis of variance and analysis of covariance. Categorical variables (stool outcomes, questionnaire and adverse event data) were compared among groups using Cochran–Mantel–Haenzsel and Fisher’s exact test. All analyses were performed using SAS^®^ Version 9.4 and SAS^®^ Enterprise Guide Version 7.1.

The period defined as “Pre-COVID-19” covered the study initiation in September 2019 up to 5 March 2020. Only 40 ITT participants completed or exited the study early during the pre-COVID-19 period. Hence, no analysis was conducted for this subset of participants alone. The period defined as “post-COVID-19” covered the period from 6 March 2020 to the end of the study. Most of the participants were in the study during the COVID-19 pandemic.

## 3. Results

### 3.1. Disposition of Participants

**Figure 2:**
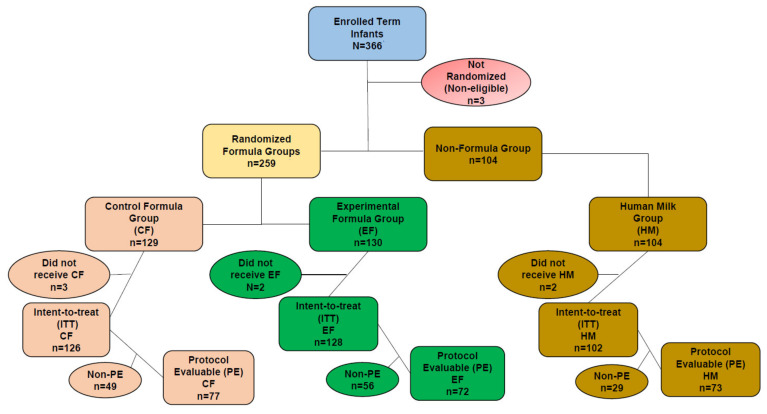
Disposition of participants.

The disposition of enrolled participants is provided in Figure 2. There were 366 participants enrolled, of whom 3 were not randomized. They consented prior to the determination of eligibility but were subsequently determined to be ineligible. Seven participants never consumed study formula and were therefore excluded from the intent-to-treat (ITT) cohort. As a result, 222 participants were included in the protocol evaluable (PE) cohort. The study completion rate on assigned feeding in the CF group was 61.9% (78/126), 59.4% (76/128) in the EF group and 83.3% (85/102) in the HM group. The rate of participants in the CF group who completed study duration was 69.0%; (87/126), 68.8% (88/128) in the EF group and 88.2% (90/102) in the HM group. A higher proportion of participants in the formula-fed groups exited the study early compared to the participants who were fed HM (all *p* = 0.009). The predominant reason for exit was lost to follow-up with 10.3% (13/126), 12.5% (16/128) and 2.9% (3/102) in the CF-, EF- and HM-fed group, respectively (EF > HM, *p* = 0.031). The mean and median ages at the D14, D28, D42, D56 and D84 visits fall within the ±3-day windows for these visits. As expected, the D119 visit has wider windows during the post-COVID-19 period; nonetheless, the mean and median ages at this visit fall within a 5-day window around D119.

Altogether, there were 26 (11.7%) PE participants with alternative anthropometric measurements at visits D28 (n = 9), D42 (n = 12), D56 (n = 1) and D84 (n = 25). Only one site elected to obtain weight measurements by parents/caregivers using Abbott-supplied digital scales calibrated by the manufacturer. Before distributing the scales, the site trained parents on their use. Alternative collection from non-study HCPs was performed for only two participants, only one of whom was PE. In the ITT cohort, 37 (10.2%) of the participants had alternative anthropometric measurements at visits D28 (n = 15), D42 (n = 20), D56 (n = 3) and D84 (n = 30).

### 3.2. Demographic and Other Baseline Characteristics

Table 1 summarizes the study population at baseline. There were no differences among the three groups for sex, age at enrollment, gestational age, birth weight, length, HC, mode of delivery, race or ethnicity, number of children < 18 years old living in the household other than the study participant, the exposure of the infant to school or daycare/group care attendance of other children < 18 years old living in the household or any family history of allergy for both ITT and PE cohorts. There was a significantly greater percentage of participants in the EF group whose mother smoked compared to percentage of participants in the CF group and in the HM group (3.9% vs 0.0%, *p* ≤ 0.022 forEF vs CF or HM groups in the ITT cohort).

### 3.3. Growth

Gompertz-predicted data from on- and off-site measurements during pre- and post-COVID-19 period are called All Gompertz. There was no significant difference in weight gain per day from D14 to D119 (which is the study primary variable and marker for growth) between the group fed EF and the group fed CF (All Gompertz PE mean ± SEM, EF: 29.4 ± 0.7 g/day and CF: 29.9 ± 0.7 g/day, *p* = 0.348; Table 2). In addition, there were no differences in weight gain per day from D14 to D119 between each study formula group and the HM reference group (All Gompertz PE, 27.9 ± 0.8 g/day, all *p* = 1.000 adjusted for multiple comparisons). The upper limit of the one-sided 97.5% confidence interval of the difference in terms of least squares means is less than 3 g, indicating that EF is non-inferior to CF. The sensitivity analysis confirmed the result of non-inferiority of EF relative to CF (using a margin of 3 g/day). There was no difference among the three groups for weight gain per day at D14-D28, D28-D42, D42-D56 and D56-D84, with one exception: males in the EF had significantly greater weight gain than males in the HM group at D84-D119 (All Gompertz PE, *p* = 0.023). There was also no difference between study formula groups for weight (repeated measures) over D14, D28, D42, D56, D84d and D119.

There were no differences in length gain from D14 to D119 between CF and EF groups in primary and sensitivity analyses (Table 2). Length gains for any interval were not different among the three groups. There was also no difference between the EF and CF groups for length (repeated measures) over D14, D28, D42, D56, D84 and D119. However, there were significant differences for length over time between HM and CF (Main effect for repeated measures, least squares mean ± SEM; All Gompertz HM: 57.5 ± 0.2 cm vs. CF: 56.6 ± 0.2 cm, *p* = 0.005) and between HM and EF groups (All Gompertz HM: 57.5 ± 0.2 cm vs. EF: 56.4 ± 0.3 cm, *p* < 0.001) from the three-group analysis in the PE group, with similar results in the ITT group. When length at study day (SD) 1 (depends on participant enrollment from 0 to 14 days of age (D)) was used as a covariate in these analyses, the differences in length between groups disappeared.

For HC gains per day from D14 to D119, there was no difference between the EF and CF groups (Table 2). The only exception was that the ITT analysis of All Gompertz indicated that EF males have lower HC gains than CF males (*p* = 0.043). Notwithstanding, primary and sensitivity analyses of the combined male and femaledata confirmed that EF was non-inferior to CF in HC gain from D14 to D119. There were no differences between EF and CF or among the three study groups in achieved HC at all timepoints for the PE cohort. For ITT’s observed data, the repeated measure analysis revealed a significant feeding-by-sex interaction indicating a difference between CF and EF among males (CF > EF, all *p* ≤ 0.030) but not among females. In the analysis of three groups, the HC gain per day at D14 to D119 in males was significantly less for the HM group than for EF (all *p* ≤ 0.048) and CF (all *p* ≤ 0.003). Interval HC gain differences between CF and EF were observed: D14-D28 (CF > EF males, *p* = 0.028, ITT), D28-D42 (CF > EF, males all *p* ≤ 0.032, ITT) and D42-D56 (EF > CF, females, *p* = 0.042, PE and CF > EF, males all *p* ≤ 0.048 ITT). There were significant differences in HC gains in males among the three groups at D42-D56 (CF > HM, all *p* ≤ 0.011, ITT), D56-D84 (CF > HM, all *p* < 0.001 and EF > HM, all *p* ≤ 0.021 PE and ITT) and at D84-D119 (EF > HM, *p* ≤ 0.035, ITT). In the PE analysis, HC gain from D84-D119 was greater in CF vs. HM (*p* = 0.023) and in EF vs. HM (all *p* ≤ 0.014).

**Figure 3:**
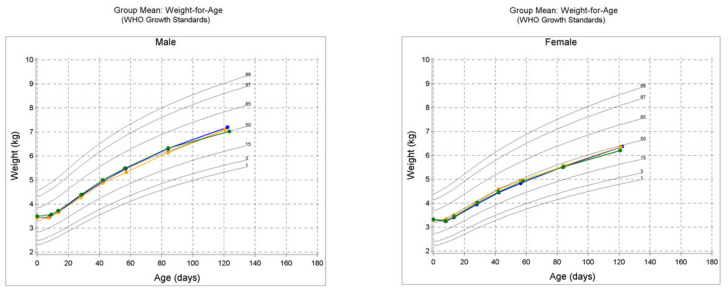

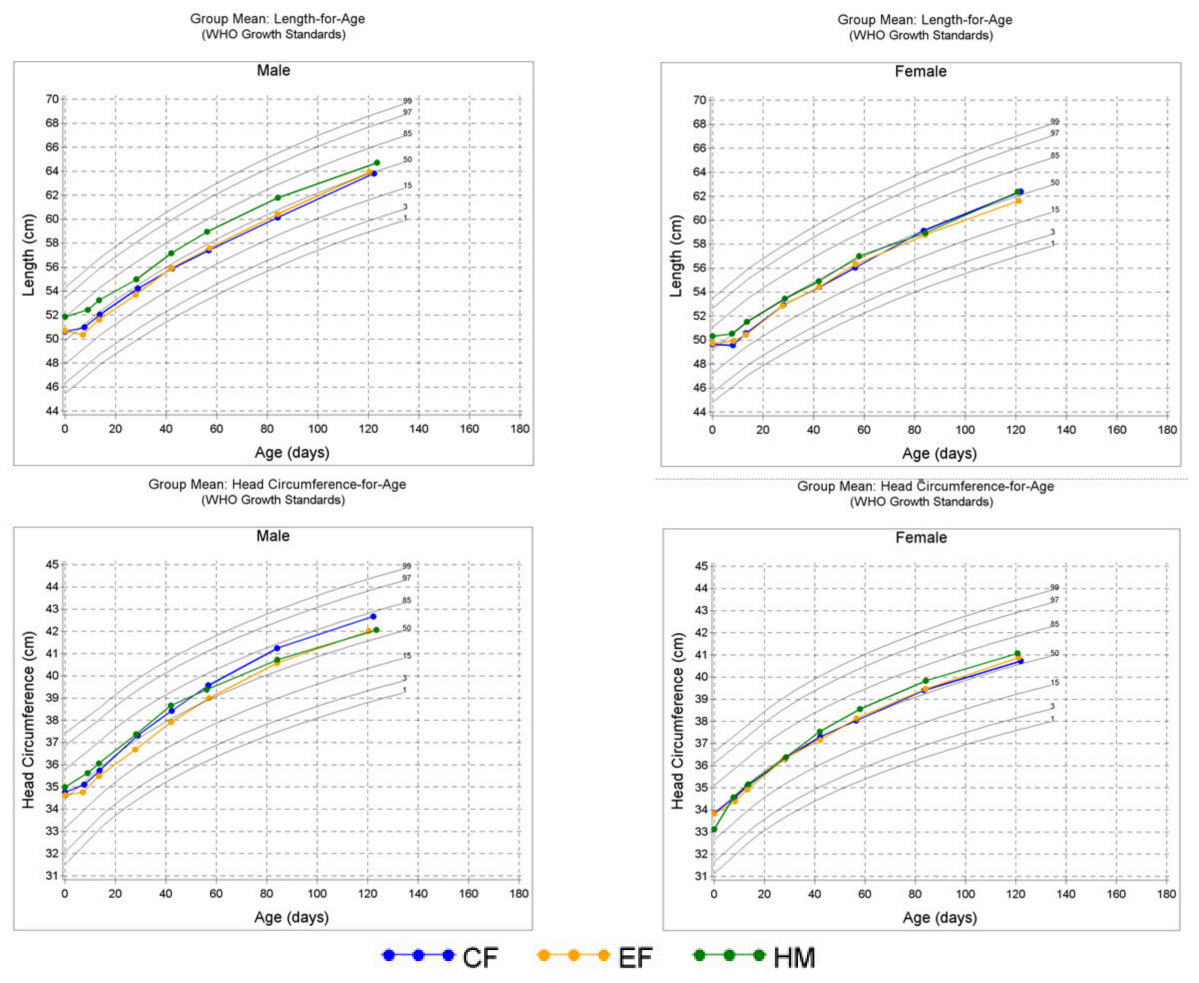
WHO growth charts for protocol evaluable (PE) male and female cohorts in the study; mean weight-for-age, mean length-for-age and mean head circumference-for-age. CF = control formula, EF = experimental formula and HM = human milk reference group.

Lastly, the growth charts presented in Figure 3 indicate that the mean weight, length and HC within each sex for the three study groups generally tracked the 50th percentile during the study.

### 3.4. Formula Intake and GI Tolerance

There were no differences noted between the formula-fed groups in intake per day and average number of feedings per day. The percentage of feedings with spit-up or vomit was not different between EF and CF in the PE cohort (Table 3); however, CF and EF were significantly greater than HM in the ITT cohorts for SD1-SD4 (*p* = 0.037 for both). For PE cohort analysis, MRSC was significantly higher (firmer) for CF than EF at D42, D56, D84, and D119 of age (all *p* ≤ 0.038), except at SD1-SD4 and SD1-D28. At D15-D28, MRSC was significantly higher for CF than EF in three-group analysis but not different in two-group analysis. In three-group PE cohort analysis, MRSC was higher for CF than HM for all timepoints (all *p* ≤ 0.006); MRSC was higher for EF than HM for SD1-SD4, D84 and D119 (all *p* ≤ 0.046); CF was higher than EF at D15-D28 (*p* = 0.047) and D42, D56, D84 and D119 (all *p* ≤ 0.015). In the ITT analysis, MRSC was significantly higher in CF than EF at all timepoints (all *p* ≤ 0.036 (two-group analysis); all *p* ≤ 0.020 (three-group analysis)); MRSC was significantly higher in CF than HM for all timepoints (all *p* < 0.001); and MRSC was higher in EF than HM at SD1-SD4 (*p* < 0.001), D84 (*p* = 0.031) and D119 (*p* = 0.003). For the two-group ITT cohort analysis, the EF group had significantly greater average number of stools per day than CF at SD1-SD4, D15-D28, D1-D28, D42, D56, D84 and D119 (all *p* ≤ 0.005) and at all timepoints except SD1-SD4 for PE (all *p* ≤ 0.004). For the three-group analysis, the HM group had significantly greater average number of stools per day than the CF or EF groups at all timepoints (all *p* ≤ 0.023 for ITT and PE), except at D119, when only the ITT HM group was greater than the CF group (*p* = 0.039). Significant differences in predominant stool consistency were noted between EF and CF groups (for two- and three-group analysis) at SD1-SD4, D15-D28 and SD1-D28 (ITT, all *p* ≤ 0.040); at D42, D56 and D84 (PE and ITT, all *p* ≤ 0.025); and at D119 (PE, *p* = 0.036), with less predominant watery stools in the CF compared to the EF group. The HM and EF groups did not differ significantly in predominant stool consistency at all timepoints except at D119 (PE and ITT, all *p* ≤ 0.018) and at SD1-SD4 (ITT, *p* = 0.015), with less predominantly watery stools in the EF group. In contrast, the HM and CF groups differed significantly at all timepoints (PE and ITT, all *p* ≤ 0.019), with CF having less predominantly watery stools compared to HM, except at D15-D28 (PE). The ITT cohort analysis revealed significant differences in predominant stool color (all *p* ≤ 0.031). The EF group had a higher percentage of infants with predominantly yellow stools compared to the CF group for SD1-SD4 (72.1% vs. 52.2%), D15-D28 (51.0% vs. 30.1%) and SD1-D28 (63.2% vs. 39.4%). In addition, the predominant yellow stools were higher in the HM group compared to the EF and CF groups (with ≥ 80% of the HM group having predominantly yellow stools across timepoints) for all comparisons in both ITT and PE cohort analyses (*p* ≤ 0.004). In contrast, the CF group had more green, brown and black predominant stools versus the EF or HM groups (Table 3).

### 3.5. Parental Responses to Study Questionnaires

Few differences were noted in the parental responses to questionnaires evaluated in this study. The most notable were the constipation dimension score and the loose stool dimension score. Parental responses indicated that the EF group had a higher average loose stool dimension score and a lower constipation dimension score compared to the CF group. The average constipation dimension is a mean score of four questionnaire items related to constipation. They included questions on if infants had at least one bowel movement per day, had difficulty moving bowels, had stools that were too hard and appeared to be constipated. The average loose stool dimension reflects the mean score of the combined three questionnaire items related to loose stools, which included if there were days infants had too many bowel movements, if infants cried or fussed before or during bowel movements and if infants had watery stools. Parents rated the questions on a five-point scale: 1 = always, 2 = frequently, 3 = sometimes, 4 = rarely and 5 = never. Because the individual questionnaire items are not in the same direction, then the individual items were reverse-scored, if needed, so that higher scores indicated poorer characteristics. The EF group had a higher average loose stool dimension score compared with the CF group at D119 (all *p* ≤ 0.033 for two-group analysis), consistent with differences in MRSC between EF and CF groups. Conversely, the CF group had higher average constipation dimension scores compared with EF and HM groups at D42, D84 and D119 (all *p* ≤ 0.074). Furthermore, parental reports indicated that the HM-fed infants wake more times in the night than infants fed either formula at D28, D42, D84 and D119 (*p* ≤ 0.011, PE). A higher proportion of infants fed CF were seen by HCPs for illness than infants fed EF from entry to D56 (11% vs. 2.9%, *p* = 0.044, PE 2-group analysis) and D84 (12% vs. 2.9%, *p* = 0.028, PE 2-group analysis) but were not different from infants fed HM at D56 and D84.

### 3.6. Safety and Adverse Events

A total of 108 (30.3%) participants had a total of 204 adverse events (AEs) including both serious AEs (SAEs) and non-serious AEs. The CF, EF and HM groups had 31.7%, 32.0% and 26.5% of participants with an AE, respectively. The majority of participants who had reported AEs experienced only one AE. A total of seven (2.0%) participants reported SAEs. This included three (2.4%) participants from the CF group, one (0.8%) participant from the EF group and three (2.9%) from the HM group. There were no significant differences (*p* = 0.249) among the participants who had SAEs between the EF and CF groups, and all but two (1 CF, 1 EF) recovered and successfully completed the study. All of the SAEs reported in infants from either formula group were medically confirmed as “not related” to the study formula by the reporting physician. There were no statistical or clinically relevant findings for SAE causality. For the non-serious AEs, there was a single statistical difference between the CF and EF groups for one system organ class (SOC), which was General disorders and Administration site conditions, (*p* = 0.029, CF = 4%, EF = 0%). However, this difference involved two preferred terms (pyrexia and irritability), both non-serious in nature, and there were no statistical differences between the groups for either of these preferred terms. AEs were assessed as mild, moderate or severe. The majority of participants who had reported AEs had maximum severity assessed as mild. Overall, there were no statistical differences among the severity assessments between the three groups. There was a difference between EF and HM for “AE highest relationship to study product” (*p* = 0.041) and between CF and HM for “PT Constipation” (*p* = 0.045). In conclusion, no safety concerns were identified with either formula group in this study.

Overall, 21% of ITT participants (26 CF, 21 EF and 28 HM) received medications during the trial. Twenty participants (seven CF, six EF and seven HM) received an antacid and/or anti-flatulent. Eighteen participants (10 CF, 4 EF and 4 HM) received topical anti-infectives. Sixteen participants received an analgesic/antipyretic, non-narcotic (four CF, four EF and eight HM). Twelve participants (four in each group) received anti-bacterial medications. Ten participants (two CF, four EF and four HM) received anti-ulcer/gastric secretion inhibitor/miscellaneous GI drugs.

## 4. Discussion

The goal of this study was to evaluate the growth and GI tolerance of healthy term infants fed an experimental infant formula (EF) containing a blend of five human milk oligosaccharides (HMOs) compared to that of infants fed a commercially representative control infant formula (CF) without supplemental prebiotics or HMOs. The CF represents a standard milk-based formula with a history of safe use in the US market. In addition, comparisons were made to a reference group of healthy term infants fed human milk (HM). The primary study variable for growth was mean weight gain per day from D14 to D119. The results of the study indicated no differences between the CF and EF groups in mean weight gain (*p* ≥ 0.05). There were also no differences in mean weight gain between the two formula groups and HM group (except at D84-D119 for male cohort). Results showed that EF was non-inferior to CF in mean weight gain per day from D14 to D119 using a non-inferiority margin of 3 g/d consistent with recommendations [39]. The observation of no difference was consistent, regardless of the methods of assessment or classification, which included protocol-evaluable (PE), intent-to-treat (ITT), study exits pre- or post-COVID-19, study exits post-COVID-19, measures collected at study sites or by alternate means, Gompertz-predicted data and observed data. Consequently, the results of the study demonstrated that growth as indicated by the mean daily weight accretion in the EF group was normal and not different from that in the CF group and the reference HM group.

Other secondary growth outcome measures assessed in this study were also supportive of no differences observed between the EF and CF groups. They include weight and interval weight gain per day, length and interval length gain per day, HC and interval HC gain per day. There were no differences noted in these measures with a few exceptions. In PE or ITT cohorts, HC gain for male participants was greater for the CF compared to the EF group at certain study timepoints. Nonetheless, both EF and CF groups were greater than the HM group in HC gain. Taken together, the current study successfully demonstrated normal growth for the EF group and were comparable to the CF and HM groups, as can be seen in the growth charts for mean weight, mean length and mean HC for both sexes (Figure 3). They tracked around or above the 50th percentile on the WHO growth chart during the study.

The observed growth of infants in our current study is consistent with findings from other studies on infant formulas with supplemental HMOs or prebiotics. Parschat et al. [22] evaluated the growth of infants fed formula supplemented with the same combination of five HMOs (2′-FL, 3-FL, LNT, 3′-SL and 6′-SL) at similar levels tested in our current study. The study concluded that the formula with the five HMOs was safe, well-tolerated and supported normal growth in healthy term infants. Other studies have shown that supplementing infant formulas with the commercially available HMOs, 2′-FL [19] and 2′-FL and LNnT [21] was safe and supported age-appropriate growth. Therefore, the results from the current study provide supplemental and complementary clinical data that safety and normal growth are not compromised when this blend of five HMOs in relative proportion and total quantity is added to infant formulas.

A secondary and supportive interest of the current study was to evaluate the GI tolerance of the EF feeding versus CF and HM feedings. The results of the current study indicated that the EF was well tolerated and was favorably comparable to the established CF formula. This is supported by the absence of significant differences (*p* ≥ 0.05) in some of the GI tolerance measures evaluated in this study, which included formula intake and occurrence of spit-up/vomit as a percentage of study feedings. Nonetheless, there were some GI tolerance measures that significantly favored EF versus CF. The EF group notably produced more frequent stools, more soft stool consistencies (MRSC, % watery plus loose/mushy stools), less firm stool consistency, more yellow-predominant stool color and less green-, brown- or black-predominant stool color compared to the CF group (Table 3). Most importantly, the EF and HM groups had no study participants with black predominant stool color. Although the HM group had more frequent stools, softer stools, yellow stools and less green or brown stools compared to the CF or EF group, the EF group had more of these stool characteristics closer to the HM group compared with the CF group. The production of frequent, softer and yellowish stools is more prominent with HM-fed infants; thus, yellow stools may be desirable for infants fed formulas as noted in some clinical studies of infants [41,42].

Multiple clinical studies have examined the impact of the use of individual HMOs in infant formula on GI tolerance in infants. Consistent with the findings in our current study, another study that assessed formula with five HMOs found that the supplemental five HMOs in the experimental formula similarly produced more frequent stools and more soft stool consistencies that were closer to HM compared to the control formula [22]. Unlike our study, the study by Parschat et al. [22] did not report an assessment of stool color. Other studies have reported on the impact of the addition of prebiotic ingredients on stool characteristics. A blend of 2′-FL and GOS added to infant formulas was well-tolerated, and these ingredients did not negatively impact infant stool frequency or stool consistency [19]. Another study that examined a formula containing 2′-FL and LNnT demonstrated significantly softer stool consistency [21]. Infant formula containing 2′-FL and FOS has been shown to have stool consistency that was similar to that seen in HM-fed infants [20,43]. The mechanism by which HMOs affect GI tolerance is most likely through providing a supply of metabolic substrates necessary for beneficial bacteria to grow and survive in the gut [1,30,44,45]. Furthermore, the production of short-chain fatty acids (SCFA) as a byproduct of HMO fermentation by the bacteria [46,47] aids in bringing the stool consistency closer to that of the breast-fed infants. In summary, the current clinical study, which comprehensively assessed GI tolerance, demonstrated that the EF containing a blend of five HMOs was well tolerated by healthy term infants.

Parental responses to questionnaires were assessed in this study as a supportive tool to evaluate GI tolerance and acceptance of the study feedings. For example, parental responses indicated that the EF group had a higher average loose stool dimension score compared with CF at D119 (all *p* ≤ 0.033), which was consistent with differences in MRSC between EF and CF groups. In contrast, parental responses indicated that the CF had a higher average constipation dimension score compared with EF and HM groups at D42, D84 and D119, which was again consistent with MRSC differences between these groups.

Although immune function markers were not evaluated in our current study nor in the study by Parschat et al. [22], it is reasonable to assume that the formulas with five HMOs can support immune functions. As discussed earlier, studies with supplemental HMOs, 2′-FL and LNnT [21] or 2′-FL and GOS [19], which were shown to support age-appropriate growth, normal GI tolerance and safe use in infants, similarly to the findings of studies with five HMOs, have also been clinically shown to support immune system benefits, including fewer reported respiratory infections and bronchitis. In addition, the findings in our current study that more infants fed CF were seen by HCPs for illness than infants fed EF from entry to D56 (*p* = 0.044, PE 2-group analysis) and D84 (*p* = 0.028, PE 2-group analysis) are supportive of the claim that the EF has immune system benefits.

The safety assessment in this study also indicated no medically significant differences between EF, CF and HM groups. The rate of 2.0% for serious adverse events (SAEs) and 30.3% for AEs including infections reported in the current study are relatively low compared to those (~46 to 70% for AEs) reported in other studies [19,22], especially for studies conducted in predominantly in periods other than the COVID-19 pandemic. The low infection rate (7.6%) and the lack of significant differences among study groups in the current study compared to the rates (~18 to 27% infection AEs) observed in other studies [19,22] could be due to multiple factors. One possible factor is that this study was conducted in a period predominated by the COVID-19 pandemic. The exposure of study participants to infectious agents during the COVID-19 pandemic period tended to be limited, possibly due to mandated quarantines, increased awareness and adherence to healthy lifestyles and diets by participants and their households. Other factors could be the differences in countries where studies are conducted and in the standard operating procedures for the collection and classification of AEs. Overall, there were no clinically relevant differences in safety measures among study groups in the current study. When taken together, these results support the conclusion that the EF formula with a blend of five HMOs can be safely fed to healthy normal infants.

The strengths of the current study include the provision of normative clinical growth data for healthy infants during the COVID-19 pandemic, as well as the inclusion of an HM group as a reference standard for growth, safety and tolerance measures. The limitation of our study is that we did not determine the amount of HM intake in the reference group because of the complexity of measuring HM intake.

## 5. Conclusions

The results from this study demonstrate that the EF with supplemental HMOs (five HMOs at 5.75 g/L) supported normal growth, tolerance and safety in healthy term infants. The growth and occurrence of AEs for the EF-fed groups were not different from the CF fed groups and the HM-fed group. Both study formulas were well tolerated; however, there were some GI measures that favored EF versus CF feeding. These include more frequent stools, loose/soft stool consistency and yellow stools that were closer to what was seen with the HM reference group.

## Figures and Tables

**Table 1:** Demographic Data for Study Participants (ITT).

	CF (*n* = 126)	EF (*n* = 128)	HM (*n* = 102)	Total (*n* = 356)
Sex, *n* (%)				
Male	62 (49.2)	62 (48.4)	49 (48.0)	173 (48.6)
Female	64 (50.8)	66 (51.6)	53 (52.0)	183 (51.4)
Ethnicity, *n* (%)				
Hispanic or Latino	25 (19.8)	26 (20.3)	25 (24.5)	76 (21.3)
Not Hispanic or Latino	100 (79.4)	102 (79.7)	77 (75.5)	279 (78.4)
Not Reported	1 (0.8)	0 (0.0)	0 (0.0)	1 (0.3)
Race, *n* (%)				
American Indian or Alaska Native	0 (0.0)	1 (0.8)	2 (2.0)	3 (0.8)
Asian	1 (0.8)	1 (0.8)	1 (1.0)	3 (0.8)
Black or African American	43 (34.1)	37 (28.9)	17 (16.7)	97 (27.2)
Native Hawaiian or Other Pacific Islanders	0 (0.0)	0 (0.0)	1 (1.0)	1 (0.3)
White	73 (57.9)	78 (60.9)	71 (69.6)	222 (62.4)
Others	9 (7.2)	11 (8.6)	10 (9.8)	30 (8.5)
Mode of Delivery, *n* (%)				
Vaginal	85 (67.5)	83 (64.8)	72 (70.6)	240 (67.4)
C-Section	41 (32.5)	45 (35.2)	30 (29.4)	116 (32.6)
Gestational age, weeks ^1^	38.8 ± 0.1	38.9 ± 0.1	38.9 ± 0.1	38.8 ± 0.1
Age at enrollment, days ^1^	7.8 ± 0.3	7.2 ± 0.3	8.0 ± 0.4	7.6 ± 0.2
Birth weight (g) ^1^	3356 ± 38	3372 ± 38	3381 ± 43	3369 ± 23
Birth length (cm) ^1^	50.2 ± 0.2	50.3 ± 0.2	50.9 ± 0.2	50.4 ± 0.1
Birth head circumference (cm) ^1^	34.2 ± 0.2	34.6 ± 0.2	34.1 ± 0.3	34.3 ± 0.1

^1^ Values are mean ± SEM. ITT = intent-to-treat, CF = control formula, EF = experimental formula, HM = human milk reference group.

**Table 2:** Anthropometric Measures (Gompertz’ predicted) ^1^.

Protocol Evaluable Cohorts (PE)		Study Groups (*n)*	D14-D119 ^2^	*p*-Values ^#^	D14-D28	D28-D42	D42-D56	D56-D84	D84-D119
Weight gain, g/day	All Pre & Post COVID-19	CF (77)	29.9 ± 0.7	0.348	39.6 ± 1.2	37.0 ± 1.0	38.9 ± 0.7	22.3 ± 0.9	22.3 ± 0.9
		EF (72)	29.4 ± 0.7		38.1 ± 1.3	35.9 ±1.1	33.1 ± 0.9	28.7 ± 0.8	22.4 ± 0.8
		HM (73)	27.9 ± 0.8		39.5 ± 1.4	36.0 ± 1.1	32.1 ± 0.9	26.5 ± 0.8	19.5 ± 0.9
	Post COVID-19	CF (72)	30.0 ± 0.7	0.337	39.7 ± 1.2	37.1 ± 1.0	34.0 ± 0.8	29.1 ± 0.8	22.5 ± 0.9
		EF (69)	29.4 ± 0.8		38.3 ± 1.3	36.0 ± 1.1	33.2 ± 0.9	28.7 ± 0.8	22.4 ± 0.8
		HM (68)	28.1 ± 0.8		39.4 ± 1.5	36.0 ± 1.2	32.3 ± 1.0	26.8 ± 0.8	19.9 ± 0.9
Length gain, cm/day	All Pre & Post COVID-19	CF (77)	0.109 ± 0.003	0.890	0.148 ± 0.006	0.131 ± 0.004	0.118 ± 0.003	0.102 ± 0.003	0.088 ± 0.005
		EF (72)	0.110 ± 0.002		0.153 ± 0.006	0.135 ± 0.004	0.120 ± 0.002	0.103 ± 0.002	0.086 ± 0.005
		HM (73)	0.103 ± 0.002		0.140 ± 0.006	0.125 ± 0.004	0.112 ± 0.003	0.098 ± 0.003	0.085 ± 0.004
	Post COVID-19	CF (72)	0.109 ± 0.003	0.918	0.150 ± 0.006	0.133 ± 0.004	0.119 ± 0.003	0.103 ± 0.003	0.087 ± 0.005
		EF (69)	0.110 ± 0.002		0.153 ± 0.006	0.135 ± 0.004	0.120 ± 0.003	0.103 ± 0.003	0.086 ± 0.005
		HM (68)	0.110 ± 0.002		0.141 ± 0.006	0.126 ± 0.004	0.113 ± 0.003	0.099 ± 0.003	0.085 ± 0.005
Head circumference gain, cm/day	All Pre & Post COVID-19	CF (77)	0.058 ± 0.001	SI ^$^	0.092 ± 0.003	0.078 ± 0.002	0.066 ± 0.002	0.052 ± 0.001	0.039 ± 0.002
		EF (72)	0.059 ± 0.002		0.089 ± 0.003	0.077 ± 0.002	0.066 ± 0.002	0.054 ± 0.002	0.042 ± 0.002
		HM (72)	0.056 ± 0.001		0.092 ± 0.004	0.076 ± 0.002	0.063 ± 0.002	0.049 ± 0.001	0.035 ± 0.002
	Post COVID-19	CF (72)	0.058 ± 0.001	0.739	0.092 + 0.004	0.078 ± 0.002	0.066 ± 0.002	0.053 ± 0.001	0.039 ± 0.002
		EF (69)	0.059 ± 0.002		0.089 ± 0.003	0.077 ± 0.002	0.066 ± 0.002	0.054 ± 0.002	0.042 ± 0.003
		HM (68)	0.056 ± 0.001		0.091 ± 0.004	0.076 ± 0.003	0.063 ± 0.002	0.049 ± 0.001	0.036 ± 0.002
Intent-to-treat Cohorts (ITT)									
Weight gain, g/day	All Pre & Post COVID-19	CF (86)	29.9 ± 0.6	0.271	39.6 ± 1.1	37.0 ± 0.9	33.9 ± 0.8	28.9 ± 0.7	22.3 ± 0.8
		EF (86)	29.5 ± 0.7		37.8 ± 1.1	35.8 ± 1.0	33.1 ± 0.8	28.9 ± 0.7	22.8 ± 0.7
		HM (86)	28.1 ± 0.7		38.7 ± 1.3	35.5 ± 1.0	32.0 ± 0.9	26.7 ± 0.7	20.3 ± 0.9
	Post COVID-19	CF (82)	30.0 ± 0.6	0.279	39.7 ± 1.1	37.2 ± 0.9	34.0 ± 0.8	29.1 ± 0.7	22.5 ± 0.8
		EF (82)	29.6 ± 0.7		38.0 ± 1.2	35.9 ± 1.0	33.2 ± 0.8	28.9 ± 0.7	22.8.0 ± 0.8
		HM (81)	28.2 ± 0.7		38.4 ± 1.3	35.4 ± 1.1	32.0 ± 0.9	27.0 ± 0.8	20.7 ± 0.9
Length gain, cm/day	All Pre & Post COVID-19	CF (86)	0.108 ± 0.002	0.708	0.147 ± 0.005	0.131 ± 0.004	0.117 ± 0.003	0.102 ± 0.002	0.087 ± 0.004
		EF (86)	0.110 ± 0.002		0.151 ± 0.005	0.135 ± 0.003	0.121 ± 0.002	0.104 ± 0.002	0.088 ± 0.004
		HM (88)	0.104 ± 0.002		0.143 ± 0.005	0.126 ± 0.003	0.112 ± 0.003	0.098 ± 0.003	0.084 ± 0.004
	Post COVID-19	CF (81)	0.109 ± 0.002	0.627	0.149 ± 0.005	0.132 ± 0.004	0.118 ± 0.003	0.102 ± 0.002	0.086 ± 0.004
		EF (82)	0.110 ± 0.002		0.151 ± 0.005	0.135 ± 0.003	0.121 ± 0.002	0.105 ± 0.002	0.089 ± 0.004
		HM (81)	0.104 ± 0.002		0.143 ± 0.005	0.126 ± 0.003	0.112 ± 0.003	0.098 ± 0.003	0.084 ± 0.004
Head circumference gain, cm/day	All Pre & Post COVID-19	CF (86)	0.038 ± 0.002	SI ^$^	0.092 ± 0.003	0.078 ± 0.002	0.066 ± 0.002	0.052 ± 0.001	0.058 ± 0.001
		EF (86)	0.043 ± 0.003		0.086 ± 0.003	0.075 ± 0.002	0.065 ± 0.001	0.054 ± 0.001	0.059 ± 0.001
		HM (88)	0.036 ± 0.002		0.090 ± 0.004	0.074 ± 0.002	0.062 ± 0.002	0.054 ± 0.001	0.055 ± 0.001
	Post COVID-19	CF (81)	0.039 ± 0.002	SI ^$^	0.092 ± 0.003	0.078 ± 0.002	0.066 ± 0.002	0.053 ± 0.001	0.058 ± 0.001
		EF (82)	0.043 ± 0.003		0.086 ± 0.003	0.075 ± 0.002	0.065 ± 0.002	0.054 ± 0.001	0.059 ± 0.001
		HM (81)	0.036 ± 0.002		0.090 ± 0.004	0.075 ± 0.002	0.063 ± 0.002	0.049 ± 0.001	0.056 ± 0.001

^1^ Values are Means + SEM (n). ^2^ D = Days of age. ^#^ *p*-values are comparing CF to EF. ^$^ SI = Significant feeding*gender interaction. There were significant differences in HC gains in males among the three groups at D42-D56 (CF > HM, all *p* ≤ 0.011, ITT), D56-D84 (CF > HM, all *p* < 0.001 and EF > HM, all *p* ≤ 0.021 PE and ITT) and at D84-D119 (EF > HM, *p* ≤ 0.035, ITT). In the PE analysis, HC gain from D84-D119 was greater in CF vs HM (*p* = 0.023) and in EF vs. HM (all *p* ≤ 0.014).

**Table 3:** Formula intake and GI stool tolerance.

		Protocol Evaluable Cohort (PE) ^1^				Intent-to-treat Cohort (ITT) ^1^			
	Study Days	Control Formula (CF)	Experimental Formula (EF)	Human Milk (HM)	*p*-Values	Control Formula (CF)	Experimental Formula (EF)	Human Milk (HM)	*p*-Values ^#^
Study formula intake, mL	D1-D28	683 ± 14 (76)	682 ± 16 (72)	NM *	0.916	662 ± 12 (102)	657 ± 13 (117)	NM *	0.864
Spit-up/Vomit as percent of feeding, %	D1-D28	13.0 ± 1.4 (76)	16.6 ± 2.9 (72)	14.4 ± 2.0 (73)	0.546	15.1 ± 1.5 (106)	16.4 ± 2.2 (118)	13.4 ± 1.6 (100)	0.973
Stool frequency, #/day	D1-D28	2.0 ± 0.2 (75)	3.0 ± 0.2 (72)	5.4 ± 0.3 (73)	<0.001	1.9 ± 0.1 (101)	3.2 ± 0.2 (117)	5.4 ± 0.2 (99)	<0.001
	D119	1.5 ± 0.1 (73)	1.9 ± 0.1 (67)	2.0 ± 0.2 (71)	0.004	1.5 ± 0.1 (76)	1.8 ± 0.1 (71)	2.1 ± 0.2 (82)	0.005
Mean rank stool consistency (MRSC), score ^2^	D1-D28	2.19 ± 0.06 (75)	2.05 ± 0.06 (72)	1.88 ± 0.06 (73)	0.177	2.27 ± 0.06 (102)	2.05 ± 0.05 (118)	1.87 ± 0.05 (100)	<0.001
	D119	2.53 ± 0.07 (73)	2.29 ± 0.08 (67)	1.99 ± 0.08 (67)	0.038	2.57 ± 0.07 (77)	2.30 ± 0.08 (71)	1.99 ± 0.07 (78)	0.036
Predominant stool consistency, n (%)	D1-D28 Watery Loose/mushy Soft Firm Hard	8 (10.8) 44 (59.5) 21 (28.4) 1 (1.4) 0 (0.0)	13 (18.3) 40 (56.3) 17 (23.9 1 (1.4)) 0 (0.0)	18 (25.0) 43 (59.7) 11 (15.3) 0 (0.0) 0 (0.0)	0.379	8 (7.9) 60 (59.4) 30 (59.4) 3 (3.0) 0 (0.0)	23 (20.0) 63 (54.8) 28 (24.3) 1 (0.9) 0(0.0)	24 (24.2) 63 (62.6) 13 (13.1) 0(0.0) 0(0.0)	0.004
Predominant stool color, n (%)	D1-D28 Yellow Green Brown Black	28 (38.4) 27 (37.0) 17 (23.3) 1 (1.4)	39 (54.9) 17 (23.9) 15 (21.1) 0 (0.0)	69 (94.5) 3 (4.1) 1 (1.4) 0 (0.0)	0.363	39 (39.4) 36 (36.4) 23 (23.2) 1 (1.0)	74 (63.2) 22 (18.8) 21 (17.9) 0(0.0)	95 (95) 4 (4.0) 1 (1.0) 0(0.0)	0.013
Average constipation dimension ^3^	D119	1.70 ± 0.06 (76)	1.52 ± 0.05 (72)	1.50 ± 0.06 (73)	0.030	1.76 ± 0.07 (84)	1.55 ± 0.05 (83)	1.50 ± 0.05 (88)	0.020
Average loose stools dimension ^3^	D119	1.76 ± 0.06 (76)	2.03 ± 0.09 (72)	1.90 ± 0.06 (73)	0.006	1.79 ± 0.06 (84)	2.0 ± 0.08 (83)	1.89 ± 0.06 (88)	0.033

^1^ Values are Means + SEM (n) except for Predominant stool consistency and Predominant stool color. ^2^ MRSC Score: 1 = Watery, 2 = Loose/mushy, 3 = Soft, 4 = Firm, 5 = Hard. ^3^ Dimension scores are parental ratings on a 5-point scale: 1 = always, 2 = frequently, 3 = sometimes, 4 = rarely, 5 = never, with higher scores indicating poorer characteristics. * Not Measured. ^#^ *p*-values are comparing CF to EF.

## Data Availability

The data are not publicly available due to privacy.
